# In Memoriam: David Judson Sencer, A Public Health Giant

**DOI:** 10.3201/eid1711.IM1711

**Published:** 2011-11

**Authors:** Jeffrey P. Koplan

**Affiliations:** Emory University, Atlanta, Georgia, USA

**Keywords:** David Judson Sencer, public health, director, Centers for Disease Control and Prevention, commentary

Many of the contributors to Emerging Infectious Diseases journal and its readers recently lost a dear friend, personally and in the field of public health. In medical school, professors often regaled us with tales of the “giants,” master diagnosticians and clinicians, physicians who could proficiently teach, investigate, care, and inspire. There is no question that David J. Sencer (Figure) was a “giant” who left his footprint (indeed, one with a worn oval in the forefoot) on many careers, institutions, and programs, and on the many people around the world who were spared illness and premature death by his efforts.

**Figure:**
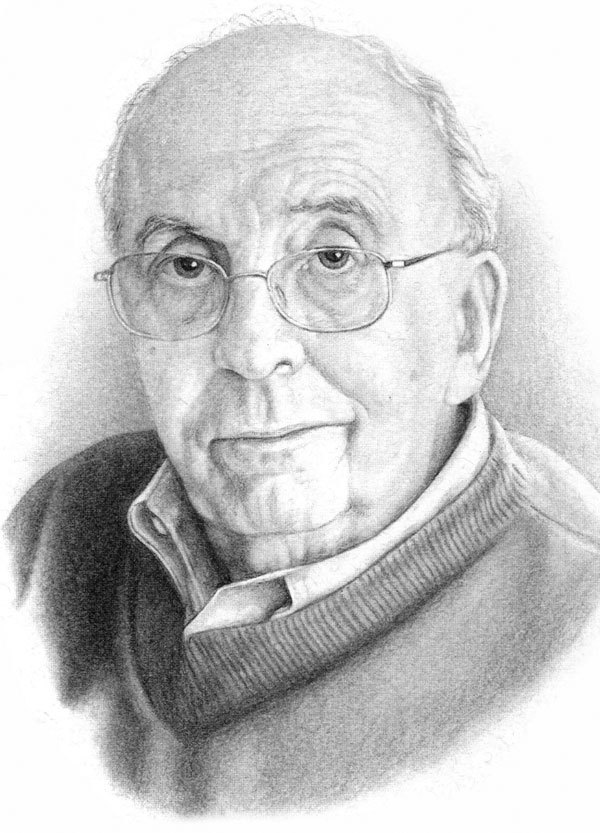
Dr. David Judson Sencer

This reflection on David Sencer is not an obituary or a biography but rather a series of observations that hope to capture his intelligence, wisdom, dedication, and humor. As he was the longest serving director of the Centers for Disease Control and Prevention (CDC) from 1966 to 1977 and one who made it his business to know virtually the whole workforce and what they did, there are many current and former CDC employees with personal accounts and recollections. Whenever possible I’ve tried to incorporate their insights and memories.

David Sencer by any measure was a great director of CDC. His accomplishments and legacy still provide a strong backbone for the agency in its structure, performance, and values. He understood that CDC had a global health mission before global health became popular and was a strong advocate for states and their primary role in our national public health system. Dr Sencer was uncompromising in the need for strong science to drive public policy. He expanded CDC’s responsibilities in keeping with the expanded mission of public health, adding nutrition, health education, and cancer epidemiology among others to a largely infectious disease portfolio. He was willing to err on the side of protecting the public’s health even if it meant a risk of critical perceptions of him and the CDC. Dr Sencer established a management system at CDC with a deep bench that was as strong as the scientific workforce. He extended CDC’s influence, strengthened our partners, and developed new skills and experiences for his staff by attaching promising and accomplished employees to state health departments, the World Health Organization (WHO), and other countries.

David Sencer was a great judge of people. He recognized talent and integrity, could see through phonies, valued colleagues who communicated with clarity and brevity, didn’t confuse science with opinion, and put the best interests of public health first above the parochial interests of their agency. He surrounded himself with talented leaders. There are far too many to name here, but David Sencer’s appointees shared his values and operating style. They gave CDC a unique personality and esprit de corps. Under Dave’s leadership, CDC grew into an agency with purpose, talent, integrity, compassion, courage, and responsiveness, and, when appropriate, an ability to laugh at itself and the ironies of public service and life’s curve balls.

David Sencer was impressive at every stage of his career, from being a young TB investigator to distinction as a CDC director and commissioner of health of New York City. But he was just as impressive in retirement. He was great fun to be with. Whatever the topic, he would be well informed, have an opinion, challenge yours, add in personal recollections and current gossip, and generate a laugh. At a dinner or public function where side commentary improved the main event, he was the person who you wanted sitting next to you. His memory was truly extraordinary. He could effortlessly embroider discussions of public health events long past with details of conversations and biographical scoop on all the players while illuminating the scientific and political elements that made the events important. He read widely, mastered the Internet, blog sites, and search engines and easily accessed archival government documents that supported his recollection of events. His emails were always informative and often offered a lesson for the recipient.

While Dave remained fully engaged in contemporary public health, he was a lifelong supporter of succession planning, long before the term was invented. He was a master mentor who never stopped mentoring. Public health workers, junior and senior, governmental and academic, who never knew him in his top leadership roles, came under his wing during his retirement. He was a frequent visitor to CDC and Emory’s Rollins School of Public Health (an institution in whose creation he played a key role) and loved attending epidemiology seminars and guest lectures. Dave remained an active public health professional throughout his life, serving as a reviewer for the Emerging Infectious Diseases journal and as active volunteer consultant on public health matters, most recently pandemic (H1N1) 2009 influenza. 

Dave enjoyed visiting people in their labs and offices and learning firsthand what they did. He did this as CDC Director and at every stage of his career, including during his “retirement,” when he would seek out students at Emory and young staff at CDC. Many young people at both institutions relished his visits and conversation and note that such an introduction often led to a long-term friendship and indeed an opportunity for Dave to mentor another promising newcomer to public health. Some topics were surefire icebreakers for Dave to initiate such conversations. They included an interest in TB or Chinese food, a connection to Michigan or New York City, and any item that would permit him to segue into a favorite anecdote. 

 A classic tale is his description, from early in his tenure as New York City Commissioner of Health, of his newfound responsibility for horses and their droppings in New York City. It is a tale worthy of the New Yorker and a most artful mix of public health and humor. It also illustrates his lifelong practice of getting advice from all quarters and addressing politics with a wise mix of pragmatism and principle. 

On one of his first days occupying the office of Commissioner of Health, he was visited by the Assistant Commissioner for Public Affairs, who told his boss that he had to be at City Hall that afternoon for a bill signing. When asked what the bill was, he was told, it was “Public Health Law No. 1,” the bill that requires the Commissioner to license carriage and riding horses in New York City. 

 Dave firmly responded, “I’m not going to do that. It’s not a health problem.”

His Assistant Commissioner replied, “Yes, you are.” 

A test of power, political reality, working relationships was unfolding as Dave tried to raise the stakes with, “I’ll go discuss it with the Mayor.” 

“No, you won’t,” said the Assistant Commissioner. 

And Dave recollected, “I didn’t.” 

 He had a canny ability to recognize the battles that needed fighting and those that didn’t, when to take advice and from whom to take it, and when to see himself both as leader and as a team member. 

David Sencer directed CDC’s growth into the linchpin of the public health system in the United States and a global force for public health. In the former, he ensured that there were balanced relationships at the local, state, and federal levels and that local and state health departments and their officials were held in respect and treated as partners by CDC, their federal counterpart. He recognized the need for CDC to grow beyond the infectious diseases and supported productive, influential, and controversial programs in family planning (now reproductive health) and environmental hazards, opening the way for research of noncommunicable diseases at CDC. He supported WHO as a global counterpart to CDC, assigning experienced CDC staff to WHO leadership roles. His support for the smallpox eradication program makes him as important as anyone assigned to the field or in Geneva for its ultimate success. As Bill Foege described in his recent history of smallpox eradication in West Africa and India (“House on Fire: the Fight to Eradicate Smallpox”), “David Sencer… always found creative ways to provide the needed equipment, people and support. It is said that genius is seeing one’s field as a whole. Sencer saw the public health world as a whole.” In all these efforts he displayed vision, the courage to make the right decision in politically hazardous situations (whether the topic was family planning and reproductive health, interventions for infected commercial products, or threats of widespread influenza) and the leadership and management skills to ensure that the efforts he started continued and ultimately were successful. 

David Sencer was such an integral part of the public health landscape that we all feel the loss of a colleague and friend. Whether we work in public health in health departments, at CDC, universities, foundations, or in industry, we are better off for his lifetime of contributions. Even more important, the people of the United States and the world are healthier because of David Sencer. He was a true giant.

